# Quinoa Polyphenol Extract Alleviates Non-Alcoholic Fatty Liver Disease via Inhibiting Lipid Accumulation, Inflammation and Oxidative Stress

**DOI:** 10.3390/nu16142276

**Published:** 2024-07-15

**Authors:** Wenjun Yao, Mingcong Fan, Haifeng Qian, Yan Li, Li Wang

**Affiliations:** State Key Laboratory of Food Science and Technology, School of Food Science and Technology, Wuxi 214026, China; 15675122924@163.com (W.Y.); fanmingcong@jiangnan.edu.cn (M.F.); qianhaifeng@jiangnan.edu.cn (H.Q.); liyan0520@jiangnan.edu.cn (Y.L.)

**Keywords:** non-alcoholic fatty liver disease, oxidative stress, AMP-activated protein kinase

## Abstract

Recently, the incidence of NAFLD has exploded globally, but there are currently no officially approved medications for treating the condition. The regulation of NAFLD through plant-derived active substances has become a new area of interest. Quinoa (*Chenopodium quinoa* Willd.) has been discovered to contain a large quantity of bioactive compounds. In this study, we established a free fatty acid (FFA)-induced steatosis model and explored the effects of quinoa polyphenol extract (QPE) on the major hallmarks of NAFLD. The results indicated that QPE significantly reduced intracellular triglyceride (TG) and total cholesterol (TC) levels. Additionally, QPE remarkably elevated the levels of superoxide dismutase (SOD), catalase (CAT) and glutathione (GSH) and lowered levels of malondialdehyde (MDA). Further examination revealed that QPE attenuated intracellular inflammation, which was verified by the reduced levels of pro-inflammatory cytokines. Mechanistically, QPE inhibited fatty acid biosynthesis mainly by targeting de novo lipogenesis (DNL) via the AMPK/SREBP-1c signaling pathway. Moreover, network pharmacology was used to analyze key targets for NAFLD mitigation by ferulic acid (FA), a major component of QPE. Taken together, this study suggests that QPE could ameliorate NAFLD by modulating hepatic lipid metabolism and alleviating oxidative stress and inflammation.

## 1. Introduction

Non-alcoholic fatty liver disease (NAFLD) is a metabolic disease identified by excessive fatty buildup in the liver induced by factors excluding alcohol consumption and other known liver-damaging factors [1]. The main features include steatosis, liver inflammation, hepatocyte vacuolation and, in severe cases, pathomorphological changes such as hepatocyte necrosis or apoptosis and liver fibrosis. NAFLD has captured widespread interest due to its increasing incidence annually. Currently, the prevalence of NAFLD hovers at approximately 25% worldwide, with rates exceeding 30% in South America and the Middle East [2]. However, there is still a lack of a specific medication to treat NAFLD in clinics. Existing drugs are mainly used to improve the metabolic syndrome caused by obesity, but the disadvantages of poor specificity and side effects make it difficult for them to be widely used. Therefore, finding effective and food-derived bioactive compounds to improve NAFLD is of great importance.

Previous studies have confirmed that hepatic lipogenesis and fatty acid β-oxidation play pivotal roles in regulating lipid deposition in the liver [3], which is linked to oxidative stress and the severity of NAFLD. Therefore, the acceleration of lipid consumption and the inhibition of lipid synthesis are considered a beneficial tactic for diminishing fat buildup in the liver. De novo lipogenesis (DNL) is a biological pathway where acetyl-CoA is transformed into fatty acids through the actions of acetyl-CoA carboxylase (ACC) and fatty acid synthase (FAS) [4]. DNL serves as a critical process in lipid metabolism, and the abnormal activation of this process may trigger hepatic steatosis. It has been reported that key lipogenic enzymes involved in DNL can be modulated at the transcriptional level by sterol regulatory element-binding protein (SREBP). Notably, SREBP-1c mainly controls triglyceride (TG)synthesis and can undergo post-transcriptional modifications by adenosine monophosphate-activated protein kinase (AMPK). AMPK is considered an important energy sensor, serving as a central enzyme protein that modulates cellular metabolism and impacts lipid metabolism balance [5,6]. Recent studies have found that many herbs or food extracts can reduce hepatic steatosis by phosphorylating and activating AMPK and inhibiting the expression of SREBP-1 [7], indicating that enhancing AMPK activity could be a useful therapeutic approach to alleviating NAFLD.

Quinoa (*Chenopodium quinoa* Willd.) is a pseudo-cereal renowned for its nutritional benefita and has been identified as a source of numerous bioactive compounds, including flavonoids, phenolic acids, saponins and polysaccharides. Although there is growing evidence that the intake of quinoa is positively associated with a reduced risk of NAFLD [8], the underlying mechanism and main biological components remain unclear [9], making breeding high-quality quinoa varieties and developing high-quality products challenging. Polyphenols extracted from quinoa have been proved to have remarkable antioxidant capacity and anti-inflammatory activity. It was previously reported that quinoa ethanol extract showed potent antioxidant activity, which was related to the high flavonoid content of the extract [10]. In a study, quinoa seeds were found to have high phenolic contents and excellent antioxidant properties based on 1,1-diphenyl-2-picrylhydrazyl (DPPH), ferric reducing antioxidant power (FRAP) and β-carotene bleaching assays [11]. An in vitro experiment also reported that quinoa polyphenols can downregulate interleukin-1β (IL-1β), IL-8 and tumor necrosis factor-α (TNF-α) cytokines in Caco-2 cells [12]. Given that oxidative stress and inflammation are the main factors in the generation and development of NAFLD, it was hypothesized that quinoa polyphenols may have an ameliorative effect on NAFLD. Although the role of quinoa polyphenols in the alleviation of NAFLD is gaining attention, studies on their specific effects are insufficient, and the molecular mechanisms involved require further investigation.

Based on this context, the current study aimed to find out the therapeutic effects of quinoa polyphenol extract (QPE) and its major phenolic acids on NAFLD. To this end, QPE was obtained by ultrasound-assisted extraction, and its phenolic composition was identified by ultraperformance liquid chromatography-coupled quadrupole time-of-flight mass spectrometry (UPLC-Q-TOF-MS). The effects of QPE on lipid accumulation, oxidative stress indicators and inflammatory cytokines were evaluated in HepG_2_ cells. Considering that AMPK regulated DNL through multiple mechanisms, we conducted an investigation into the changes in AMPK and its downstream substrates before and after QPE treatment in the cells. Moreover, a network pharmacology analysis was performed to determine the potential targets and molecular mechanisms of the main phenolic acids in QPE to alleviate NAFLD. Our results may expand the active functions of quinoa and meet the demand for effective dietary supplements to ameliorate NAFLD.

## 2. Materials and Methods

### 2.1. Materials

Black quinoa seeds were kindly provided by the zonlife company (Shuozhou, China). Quinoa seeds were ground and sieved through 60 mesh, and quinoa powders were stored in polyethylene bags sealed at −20 °C until use. Phenolic acid standards were obtained from Macklin Inc. (Shanghai, China). HPLC-grade acetonitrile and formic acid were purchased from Merck (Darmstadt, Germany). Kits for measuring total cholesterol (TC) and triglyceride (TG) were acquired from the Jiancheng Bioengineering Institute (Nanjing, China). The bicinchoninic acid (BCA) protein quantification kit, HiScript^®^ III RT SuperMix for the qPCR reverse transcription kit and ChamQ Universal SYBR qPCR Master Mix kit were provided by Vazyme Biotech Co., Ltd. (Nanjing, China). Modified Dulbecco medium (DMEM) and fetal bovine serum (FBS) were purchased from Thermo Fisher Scientific Inc. (Waltham, MA, USA). Sodium oleate and sodium palmitate for cell experiments were purchased from Mreda (Beijing, China). FFA-free bovine serum albumin (BSA) was purchased from Solarbio (Beijing, China). Antibodies against FAS, AMPK, phosphorylated AMPK (*p*-AMPK) and GAPDH were purchased from Cell Signaling Technology (Danvers, MA, USA). Anti-SREBP-1c was purchased from Santa Cruz Biotechnology (Dallas, TX, USA). Omni-ECL Femto Light Chemiluminescence kit was purchased from Epizyme Biotech (Shanghai, China). Human hepatoma HepG_2_ cells were collected from the cell bank of the Chinese Academy of Sciences (Shanghai, China).

### 2.2. Extraction of QPE

The quinoa powders were degreased by stirring with hexane and evaporated to dryness. A total of 100 g of quinoa powders was extracted with 1 L of 80% acetone in an ultrasonic bath (KQ-500ES, Kunshan, China) operating at a frequency of 40 kHz for 30 min. The extract was centrifuged (Hitachi CR-GIII, Hitachi Ltd., Tokyo, Japan) for 15 min at 4 °C and 8000× *g*. The extraction was repeated twice, and the resulting supernatants were combined. After adjusting the pH to 1.5–2.0, the extract was concentrated at 45 °C using a rotary evaporator (BUCHI R-300, BUCHI Ltd., Flawil, Switzerland). The extract was then extracted 2–3 times with ethyl acetate and was concentrated at 37 °C. Finally, the extract was re-dissolved with ultrapure water and was lyophilized in a lyophilizer (LGJ-10E, Sihuan Ltd., Beijing, China). The lyophilized samples were subsequently stored in a −20 °C freezer until use.

### 2.3. UPLC Q-TOF-MS Analysis of Phenolic Compounds in QPE

The separation by chromatography was carried out on a Waters ACQUITY UPLC system (Waters, Milford, MA, USA) equipped with a BEH C18 analytical column (2.1 mm × 100 mm, 1.7 μm). The mobile phase consisted of (A) acetonitrile and (B) 125 mM formic acid with the following gradient: 0–0.5 min, 2% A; 0.5–10 min, 2–20% A; 10–18 min, 20–100% A; 18–21 min, 100% A; 21–21.5 min, 100–0% A. The injection flow rate was 0.3 mL/min, and the column temperature was 40 °C. The mass spectrometry data acquired by the Q-TOF-MS detector (Waters, MA, USA) were used to detect phenolic substances in the QPE. The electrospray was set to negative ion mode with a mass scan range of *m*/*z* 50–1200. The capillary voltage was 3 kV, while the cone voltage was 40 V. Nitrogen (N_2_) and helium (He) were selected as the desolvation gas and cone gas, with flow rates of 800 L/h and 50 L/h, respectively. A data analysis was conducted with the help of MassLynx v4.2 software. The identified compounds were quantified using standard substances.

### 2.4. Cell Culture and Treatment

HepG_2_ cells were cultured in complete medium (90% DMEM, 10% FBS, 1% Penicillin-Streptomycin solution). Cells were placed in an incubator set at 37 °C with 5% CO_2_. At the stage of 80–90% cell fusion, the cells were digested and passaged for subsequent experiments. The steatosis HepG_2_ cell model was induced with FFA (sodium oleate/sodium palmitate = 2/1) to stimulate excessive lipid synthesis according to the previously reported method [13]. HepG_2_ cells were exposed to varying concentrations of FFA and QPE in a medium supplemented with 1% FFA-free BSA for 24 h. The control group was complete medium containing a final concentration of 1% FFA-free BSA.

### 2.5. Cell Viability

The cell viability was determined by MTT assay. Briefly, HepG_2_ cells were seeded into 96-well plates at 1 × 10^5^ cells/well and then exposed to different concentrations of FFA (0, 150, 300, 450, 600, 750, 900 μmol/L) and QPE (0, 10, 20, 40, 80, 160, 320, 640, 1280, 2560 µg/mL) for 24 h. Next, 10 µL of MTT solution (Merck KGaA, Darmstadt, Germany) was added to each well and allowed to incubate for 4 h. After that, the cells were incubated with 150 µL of DMSO solution, and the absorbance was measured at 490 nm using a microplate reader (BioTek Ltd., Winooski, VT, USA). Each treatment was replicated three times.

### 2.6. Oil Red O Staining

The cell-staining process was performed as previously described [14]. In brief, HepG_2_ cells were immobilized by 4% formalin for 30 min and stained with Oil Red O reagent for 30 min at 4 °C. The cells were rinsed with sterile water 3–4 times to clear excess staining solution. The washed plates were then observed under a fluorescent inverted microscope (Axio Vert A1, Carl Zeiss AG, Oberkochen, Germany) and photographed. After observation, isopropanol was added to the plates, and the eluates were used for the quantification of Oil Red O content at a wavelength of 490 nm.

### 2.7. Measurement of Intracellular TC and TG

After treatments, cell lysis solution (2% Triton X-100 lysis for 30 min) was added to the cell sediment to completely lyse the cells. The concentration of proteins was determined by BCA assay, and the intracellular TC and TG contents were quantified according to the methods described in the TC and TG kit instructions.

### 2.8. Measurement of Oxidative Stress

Oxidative stress can be detected by measuring intracellular superoxide dismutase (SOD), malondialdehyde (MDA), glutathione (GSH) and catalase (CAT) levels. Measurements were obtained using commercial kits purchased from Solarbio Co. (Beijing, China).

### 2.9. Real-Time Quantitative Polymerase Chain Reaction (RT-qPCR) Analysis

Total cellular RNA was obtained with Trizol reagent (Takara Bio, Beijing, China) and converted to cDNA following the kit instructions. An amount of 4.6 μL of cDNA was used in each qPCR reaction, and the number of qPCR cycles was 40. The implementation of the PCR analysis was conducted on the Applied Biosystems QuantStudio™3 (Thermo Fisher Scientific Inc., Waltham, MA, USA) with the employment of QuantStudio™ design and analysis software. The PCR primers were synthesized by Shangya Biotechnology Co., Ltd. (Shanghai, China), and their sequences can be found in Table S1 of the Supplementary File. The calculation of relative gene expressions was determined by the method of 2^−△△Ct^ with the use of GAPDH as the internal reference gene.

### 2.10. Western Blot Analysis

A total of 1 × 10^6^ HepG_2_ cells were seeded in 6-well plates. After treatment, the cultured cells were collected and lysed. Next, the extracted protein samples were separated by 10% SDS-PAGE electrophoresis and subsequently transferred onto PVDF membranes. The membranes were blocked with 5% skim milk for 1 h and washed with Tris-buffered saline (TBS)–Tween. After completion of the blotting and blocking steps, membranes were incubated with one of the following primary antibodies at 4 °C overnight: anti-FAS (1:1000, 3180), anti-SREBP-1c (1:1000, sc365513), anti-*p*-AMPK (1:1000, 2535), anti-AMPK (1:1000, 2532) and anti-GAPDH (1:4000, 97166), followed by another 1 h incubation with an appropriate secondary antibody. Finally, the optical density values of the protein bands were measured with ImageJ 1.54 software and normalized to GAPDH.

### 2.11. Network Pharmacology Study

#### 2.11.1. Identification of Potential Targets of Ferulic Acid (FA) and Protocatechuic Acid (PCA)

The target prediction of FA and PCA was performed using SwissTargetPrediction (www.SwissTargetPrediction.ch, accessed on 18 October 2023), PubChem (https://pubchem.ncbi.nlm.nih.gov/, accessed on 18 October 2023) and Pharm Mapper (http://www.lilab-ecust.cn/pharmmapper/, accessed on 19 October 2023). The results were standardized with the help of the UniProt database (https://www.uniprot.org/, accessed on 19 October 2023).

#### 2.11.2. Collection of Core Targets of NAFLD

The targets associated with NAFLD were collected from the GeneCards database (www.genecards.org, accessed on 20 October 2023) and the OMIM database (https://omim.org/, accessed on 20 October 2023).

#### 2.11.3. Construction of the Protein–Protein Interaction (PPI) Network

With the aim of exploring the possible mechanism between the two phenolic compounds and targets related to NAFLD, we integrated the relevant target information into the STRING database (https://string-db.org, accessed on 26 October 2023). Subsequently, the interaction networks between these targets were mapped and visualized with the help of Cytoscape 3.9.1 software. The Centiscape plug-in was then employed to compute the degree and betweenness of each gene in the network. Targets were labeled as key if their degree and betweenness values were equal to or higher than the average.

#### 2.11.4. Gene Ontology (GO) and Kyoto Encyclopedia of Genes and Genomes (KEGG) Pathway Enrichment Analysis

The collected intersection targets of the phenolic compounds and NAFLD were entered into the DAVID database (https://david.ncifcrf.gov/, accessed on 26 October 2023). GO analysis is a powerful tool to systematically describe the function of genes and their protein products. It involves three main levels: molecular function (MF), biological process (BP) and cellular component (CC) analyses. The enriched terms of BP, MF and CC were sorted according to *p*-value, and the top 20 terms were filtered and plotted as bubble charts. In addition, the KEGG pathway enrichment analysis is a key technique for exploring potential signaling pathways related to the candidate targets in the improvement of NAFLD.

### 2.12. Molecular Docking

The top eight proteins were identified as receptors and FA molecules as ligands. The 3D structures of the proteins were firstly obtained from the Protein Data Bank (PDB) (https://www.rcsb.org/, accessed on 1 June 2024). The MOL2 format of FA was obtained from the TCMSP database (https://old.tcmsp-e.com/tcmsp.php, accessed on 1 June 2024) and was converted to the PDB format by Autodock. Water removal and hydrogenation were performed using AutodockTools 1.5.7 software, and the results were saved in PDBQT format. The docking box coordinates were then determined and molecular docking operations were performed using Autodock vina software. Finally, pymol 2.1.0 was used for visualization to obtain 3D analytical charts.

### 2.13. Statistical Analysis

SPSS 26.0 software and GraphPad Prism 9.0 were employed to conduct a statistical analysis. Experimental data were reported as mean ± standard deviation (SEM) from a minimum of three independent experiments. The statistical analysis was examined with a one-way analysis of variance (ANOVA), followed by Fisher’s least significant difference (LSD) test. *p* < 0.05 was deemed to be statistically significant.

## 3. Results

### 3.1. Polyphenolic Composition of QPE

The phenolic composition of the QPE was analyzed by UPLC Q-TOF-MS. As revealed in Table 1, seven phenolic acids were successfully identified based on the mass spectrometry data of these substances, specifically, vanillic acid, PCA, caffeic acid, *p*-hydroxybenzoic acid, *p*-coumaric acid, FA and sinapic acid. In addition, two flavan-3-ol monomers, catechin and epicatechin, were detected in the QPE. The identified compounds were quantitatively analyzed by comparison with standard substances (see Table S2 for details). The results of the study showed that the main phenolic substances with a high content in QPE were PCA and FA. As reported, PCA was the dominant phenolic acid both in red and black quinoa, and black quinoa had the highest phenolic acids concentration among white, red and black quinoa [15]. A recent study also reported that black and red grains had markedly higher contents of *p*-coumaric acid and FA [16]. These results were consistent with our study.

### 3.2. QPE Attenuated FFA-Induced Lipid Accumulation in HepG_2_ Cells

**Figure 1:**
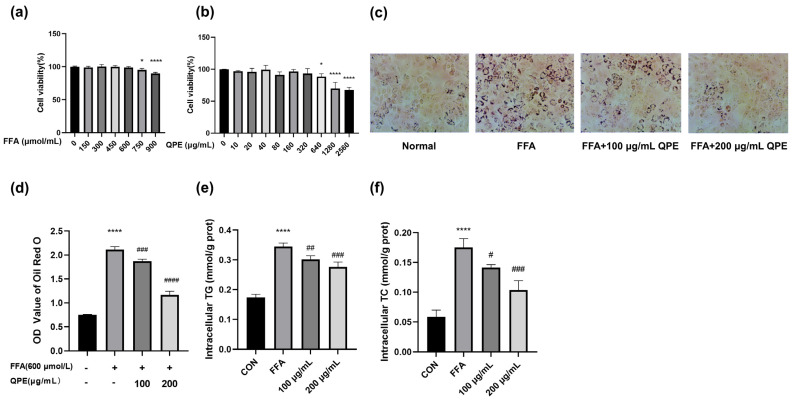
(**a**) Effect of FFA on cell viability. (**b**) Effect of QPE on cell viability. (**c**) Oil red O staining microscope photographs taken for normal control, FFA- and QPE-treated groups at 200× magnification. (**d**) Lipid accumulation was quantified by colorimetric method. (**e**,**f**) Effect of QPE on intracellular TG and TC levels. Data are presented as means ± SD (*n* = 3), * *p* < 0.05, **** *p* < 0.0001 compared to control group and # *p* < 0.05, ## *p* < 0.01, ### *p* < 0.001, #### *p* < 0.0001 compared to model group.

Prior to performing the experiments, we screened the FFA concentrations. Up to 600 μM, the MTT results confirmed that FFA did not have any cytotoxic effects (Figure 1a). Therefore, 600 μM of FFA was selected as the modeling concentration in the following experiments. Subsequently, HepG_2_ cells were subjected to QPE at different concentrations (0, 10, 20, 40, 80, 160, 320, 640, 1280, 2560 μg/mL) to detect cell viability. As shown in Figure 1b, the proliferation of HepG_2_ cells was significantly decreased after treatment with 640 μg/mL of QPE, suggesting that the concentrations of QPE used in this experiment (100 μg/mL and 200 μg/mL) were safe. In addition, Oil Red O staining was used to visualize the impact of QPE on FFA-induced lipid deposition, and the relative lipid accumulation was calculated by quantifying the OD value at 490 nm. The results revealed a significant rise in lipid droplets in the model cells as opposed to the control group, with QPE notably decreasing FFA-induced lipid deposition (Figure 1c,d). Consistently, the TG and TC levels in the model group were markedly elevated compared with the control group, indicating the successful establishment of a steatosis cell model by stimulating cells with 600 μM FFA. A significant reduction in TG and TC contents was observed after QPE treatment, as opposed to the FFA group (Figure 1e,f). The findings showed that QPE was successful in lowering intracellular TG and TC levels and reducing lipid accumulation in HepG_2_ cells.

### 3.3. QPE Attenuated Oxidative Stress Caused by Lipid Accumulation in HepG_2_ Cells

**Figure 2:**
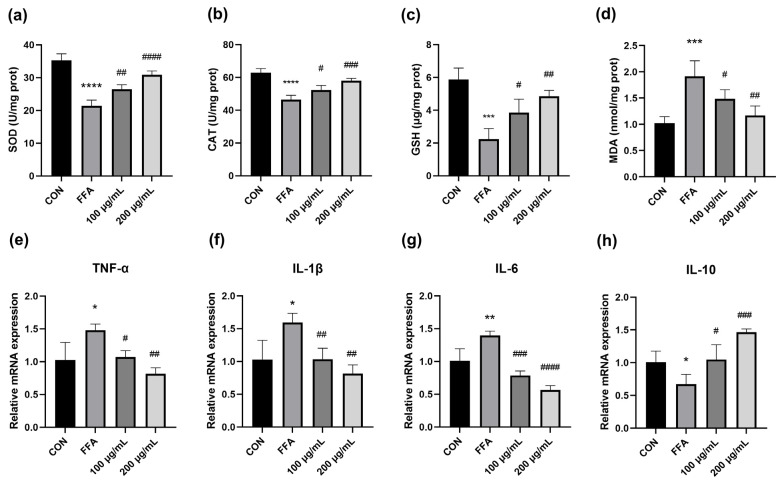
The evaluation of oxidative stress and inflammation in HepG_2_ cells treated with QPE. (**a**,**b**) The activity of SOD and CAT. (**c**) The GSH content. (**d**) The MDA content. (**e**–**h**) The relative mRNA expressions of TNF-α, IL-1β, IL-6 and IL-10. Data are presented as means ± SD (*n* = 3), * *p* < 0.05, ** *p* < 0.01, *** *p* < 0.001, **** *p* < 0.0001 compared to control group and # *p* < 0.05, ## *p* < 0.01, ### *p* < 0.001, #### *p* < 0.0001 compared to model group.

Oxidative stress occurs when there is an imbalance between the oxidative and antioxidant systems of cells and tissues, leading to an overproduction of oxygen radicals and reactive oxygen species (ROS). To evaluate the extent of oxidative stress in cells after liver injury, an evaluation of MDA, SOD, CAT and GSH levels in HepG_2_ cells was conducted. In comparison to the untreated cells, the antioxidant enzymes were downregulated, and the MDA content was significantly elevated in the model group, indicating that FFA-induced excessive oxidative stress occurred in the HepG_2_ cells. Treatment with QPE remarkably increased the activities of SOD and CAT and the GSH content (Figure 2a–c). Meanwhile, the QPE groups displayed a significant decline in MDA content compared with the model group (Figure 2d). This suggested that lipid buildup exacerbated oxidative stress in HepG_2_ cells, while QPE was found to notably alleviate oxidative stress.

### 3.4. QPE Alleviated FFA-Induced Inflammatory Response

Several studies have confirmed a relationship between NAFLD and the release of pro-inflammatory cytokines [17,18]. To delve deeper into the impact of QPE on the inflammatory response, we evaluated the levels of several major inflammatory cytokines. Interleukin-1β (IL-1β), IL-6 and tumor necrosis factor-α (TNF-α) are pro-inflammatory factors secreted by macrophages and are positively correlated with the level of inflammation. Compared to the untreated cells, the FFA-induced group exhibited a considerable increase in relative mRNA levels of IL-1β, IL-6, and TNF-α, while the levels of these elevated pro-inflammatory cytokines were significantly lowered after QPE intervention (Figure 2e–g). Notably, QPE remarkably upregulated the expression of mRNA for IL-10 compared to the model group (Figure 2h). By inhibiting pro-inflammatory cytokine synthesis and blocking NF-κB activity, IL-10 serves as an important anti-inflammatory mediator [19]. The combined findings demonstrated that QPE has the potential to effectively alleviate NAFLD through the regulation of oxidative stress and inflammation.

### 3.5. Effect of QPE on mRNA Expression of Lipid Metabolism-Related Enzymes in NAFLD Cell Models

**Figure 3:**
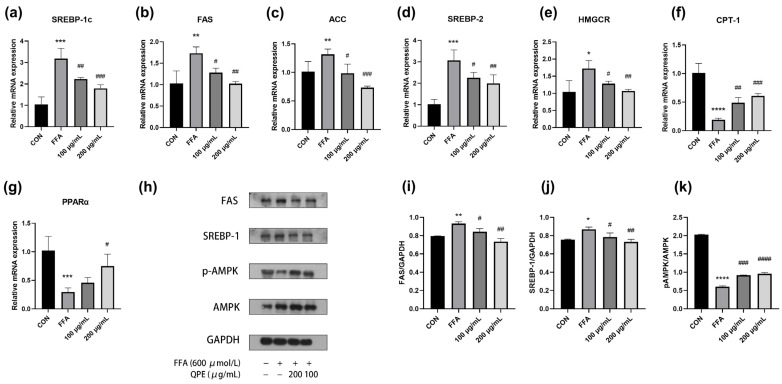
(**a**–**g**) RT-PCR was conducted to detect relative mRNA expressions of genes responsible for lipid metabolism in HepG_2_ cells. (**h**) The protein expressions of FAS, SREBP-1c, *p*-AMPK and AMPK in HepG_2_ cells were detected by Western blot. (**i**–**k**) The gray analysis was analyzed by ImageJ 1.54 software. Data are presented as means ± SD (*n* = 3), * *p* < 0.05, ** *p* < 0.01, *** *p* < 0.001, **** *p* < 0.0001 compared to control group and # *p* < 0.05, ## *p* < 0.01, ### *p* < 0.001, #### *p* < 0.0001 compared to model group.

In order to explore how QPE alleviates NAFLD, we studied the gene levels responsible for lipid metabolism in HepG_2_ cells. The relative mRNA expressions of SREBP-1c, FAS, ACC, SREBP-2 and 3-hydroxy-3-methylglutaryl-CoA reductase (HMGCR) in the FFA group were significantly increased, whereas the relative mRNA expressions of carnitine palmitoyltransterase-1 (CPT-1) and peroxisome proliferators-activated preceptor α (PPARα) were significantly lower in the FFA group than in the control group (Figure 3a–g). As shown in (Figure 3a–c), QPE significantly downgraded the mRNA levels of the SREBP-1c, FAS and ACC genes responsible for DNL compared to the model group. QPE also significantly increased the mRNA levels of the CPT-1 and PPARα genes involved in fatty acid β-oxidation (Figure 3f,g). Altogether, these results suggest that QPE decreased the excessive lipid buildup by upregulating the mRNA expressions of lipolysis-related genes and downregulating the mRNA expressions of lipogenesis-related genes. Moreover, the influence of QPE on AMPK activity was assessed. Immunoblotting studies demonstrated that QPE reversed the reduction in phosphorylated AMPK, which in turn decreased the protein expression levels of SREBP-1c and FAS, leading to the inhibition of TG synthesis in HepG_2_ cells (Figure 3h,k).

### 3.6. Target Network Analysis

**Figure 4:**
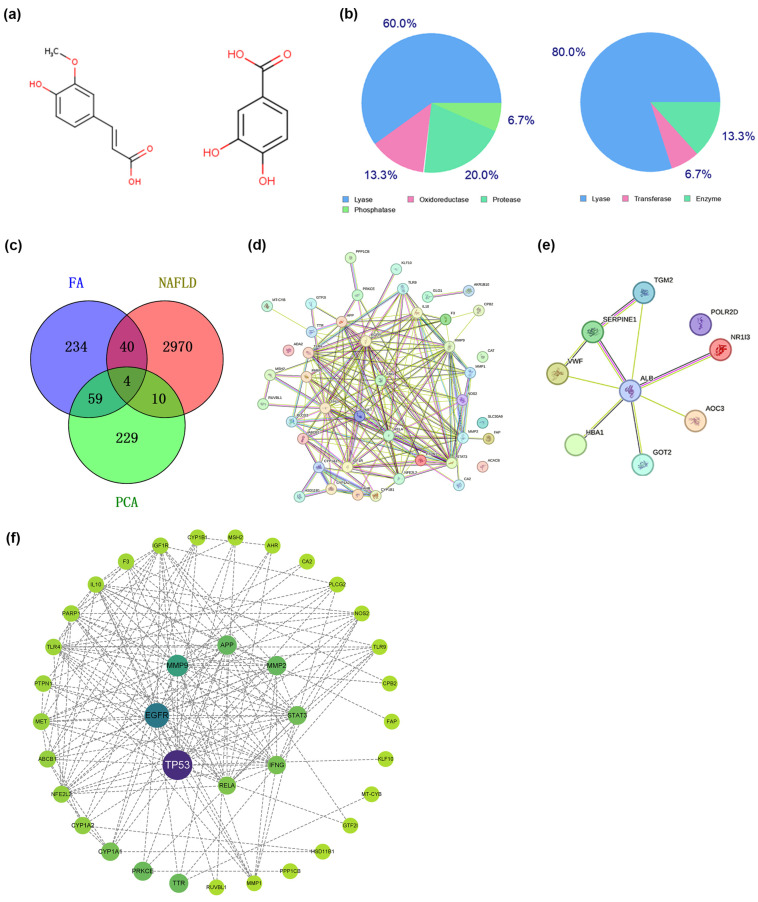
Predicted effects of phenolic acids against NAFLD. (**a**) Chemical structures of FA and PCA. (**b**) Target classes of FA and PCA. (**c**) Venn diagram of FA and PCA target genes crossed with genes related to NAFLD. (**d**) The PPI network of FA target genes. (**e**) The PPI network of PCA target genes. (**f**) The PPI network for FA acting on NAFLD. The target network consisted of 37 nodes and 172 edges, with the nodes symbolizing the targets of FA acting on NAFLD and the edges denoting relationships between the targets.

Considering that FA and PCA were the two main phenolic acids in QPE, the present study preliminarily studied the molecular mechanism of FA and PCA alleviating NAFLD by network pharmacology. By analyzing the structures of FA and PCA (Figure 4a), their target genes were predicted using SwissTargetPrediction and the Pharm Mapper database, and a total of 234 FA and 229 PCA target genes were obtained. A Venn diagram was constructed to obtain 40 common targets of FA and NAFLD, and 10 common targets of PCA and NAFLD (Figure 4c). The Cytoscape plug-in PPI network, based on the STRING database and topological data analysis, was used to construct a PPI network that had 37 connected nodes and 172 relationship pairs (Figure 4e). The network analysis showed that the degree average value and the betweenness average value were 9.30 and 36.05, respectively. Targets with an above-average degree and betweenness were regarded as key targets that may have important roles in regulating NAFLD-related biological processes [20]. The eight key targets meeting this selection criterion, listed in descending order of betweenness (Table S3), were tumor protein p53 (TP53), epidermal growth factor receptor (EGFR), matrix metalloproteinase-9 (MMP9), amyloid beta precursor protein (APP), MMP2, signal transducer and activator of transcription 3 (STAT3), interferon gamma (IFNG) and NF-κB subunit p65 (RELA).

### 3.7. Pathway Enrichment Analysis of FA Target Genes

**Figure 5:**
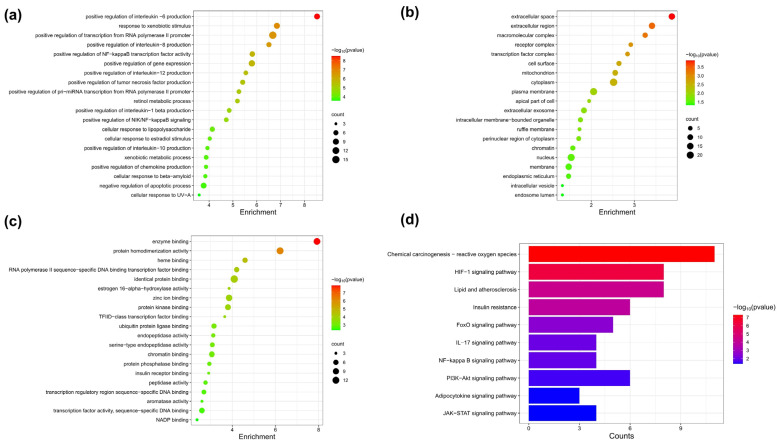
Functional enrichment analysis. (**a**) The BP analysis. (**b**) The CC analysis. (**c**) The MF analysis. (**d**) The KEGG enrichment analysis.

Common targets were analyzed by employing the GO and pathway enrichment analysis functions within the DAVID database. The top 20 enriched terms of BP, MF and CC were selected based on *p*-value and screened to draw dot bubble charts. The BP analysis revealed that FA acts mainly through the positive regulation of interleukin-6 production, positive regulation of gene expression and positive regulation of NF-κB transcription factor activity (Figure 5a). The CC analysis revealed that the targets are mainly associated with the cytoplasm, mitochondrion and plasma membrane (Figure 5b). Moreover, the MF analysis indicated that FA exerted its function by interacting primarily with enzyme binding, protein homodimerization activity, identical protein binding, protein kinase binding transcription factor activity and sequence-specific DNA binding (Figure 5c). Based on the KEGG enrichment analysis, it could be seen that the alleviating effect of FA on NAFLD mainly involved ROS, the HIF-1 signaling pathway, lipids and atherosclerosis, insulin resistance, and the PI3K-Akt signaling pathway, which mediated the potential mechanism of FA intervention in NAFLD (Figure 5d).

### 3.8. Molecular Docking of FA with Core Targets

**Figure 6:**
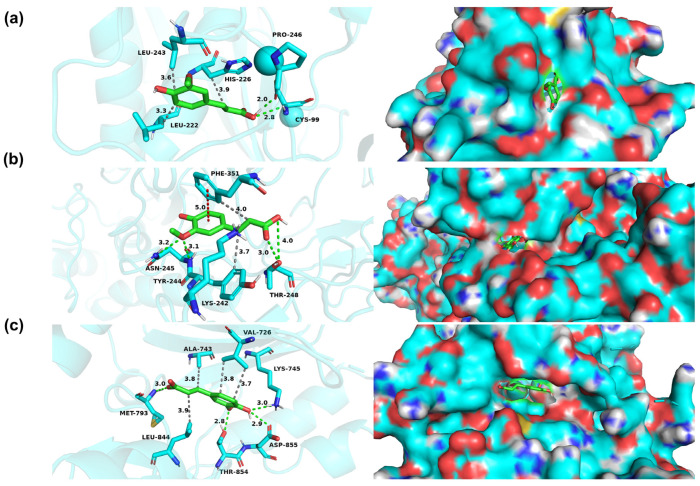
Visualization of molecular docking. (**a**) MMP9. (**b**) MMP2. (**c**) EGFR.

To further verify the accuracy of the core targets (APP, EGFR, IFNG, MMP2, MMP9, RELA, STAT3 and TP53) obtained in the network, a molecular docking analysis between core targets and the FA molecule was performed in Autodock vina (Table 2). According to the binding energy, the top three target proteins (MMP9, MMP2 and EGFR) were selected for visual analysis (Figure 6). Figure 6 showed the non-bonding interactions, including hydrogen bonding (indicated by the green dashed line), the π-π stacking effect (indicated by the red dashed line) of the FA molecule with the surrounding amino acid residues, hydrophobic interactions (indicated by the gray dashed lines) and the space between the FA molecule and the protein pocket position.

## 4. Discussion

NAFLD is now recognized as a prevalent and rising liver disease globally, although its pathogenesis is currently unclear. Hepatic steatosis is an indispensable initiator of NAFLD and has a profound impact on the pathology of NAFLD. The Oil Red O staining results clearly indicated a notable rise in intracellular lipid levels in the model group and a significant decrease in intracellular lipid content following QPE treatment, which implied that QPE might lessen the lipid accumulation induced by FFA (Figure 1a–c). The results also showed that QPE intervention greatly reduced the intracellular TG and TC contents increased by FFA (Figure 1d,e). Therefore, it can be said that QPE shows significant physiological lipid-lowering activity.

Oxidative stress plays a crucial role in the occurrence and progression of NAFLD by impairing mitochondrial function, causing an imbalance between intracellular lipid synthesis and catabolism and ultimately leading to cellular steatosis. The degree of oxidative stress correlates significantly with the severity of NAFLD, and an abnormal expression of oxidative stress-related markers is frequently present in the liver of NAFLD patients. Previous studies reported that quinoa seeds slowed down fat peroxidation, effectively reduced plasma MDA concentrations and maintained SOD, CAT and GPx activities in the normal range [21]. Researchers found that red and yellow quinoa sprout extract was effective in reducing MDA and enhancing GSH and SOD activities in rats. This result suggested that the phenolic and flavonoid components contained in red sprouts may have potent bioactivities in attenuating oxidative stress, as well as hepatic inflammatory responses [22]. Our research showed that QPE significantly improved the activity of SOD, CAT and GSH in HepG_2_ cells (Figure 2a–c). When the body undergoes oxidative stress, a large number of free radicals are generated, leading to the oxidation of fatty acids and their breakdown into a series of lipid peroxides such as MDA. MDA generated through lipid peroxidation can damage cell membranes and lead to impaired cell function, making it a key indicator of the severity of cellular damage. SOD is the main antioxidant enzyme responsible for the scavenging of superoxide anion, which is essential for protection against oxidative-induced injuries and inflammatory reactions. As mentioned above, the increase in SOD, CAT and GSH activity coupled, with the decrease in MDA, after QPE indicated an alleviation of oxidative stress, thereby improving NAFLD.

Elevated levels of IL-1β, IL-6 and TNF-α were found to be strongly correlated with an increased risk of NAFLD in a meta-analysis [15]. TNF-α not only promotes inflammation itself, but also exacerbates the inflammatory reaction by inducing the synthesis of other inflammatory cytokines. Previous studies have found that IL-10, a key anti-inflammatory cytokine, can efficiently inhibit the release of various inflammatory cytokines by T cells [23]. Upon investigation, it was observed that QPE remarkably decreased the mRNA levels of IL-1β, IL-6 and TNF-α, while increasing the mRNA expression of IL-10 (Figure 2e–h). Collectively, the present study indicated that QPE might have a positive influence on alleviating NAFLD by decreasing lipid accumulation, relieving oxidative stress and regulating inflammatory cytokines. Therefore, we further investigated the molecular mechanisms involved.

Hepatic fat accumulation is viewed as a major contributor to the development of NAFLD and can be triggered by several causes, including elevated hepatic FFA absorption, impaired fatty acid β-oxidation and elevated DNL. Considering DNL is a complex regulatory process, the regulation of pivotal nuclear transcription factors and enzymes in DNL is critical for the control of lipid metabolism. As a key enzyme in fatty acids synthesis, ACC regulates the production of malonyl-CoA, which is an allosteric inhibitor of CPT1 [24]. Previous studies suggest that resveratrol ameliorated NAFLD induced by high-fat diets, and this effect was associated with a reduction in the hepatic mRNA expressions of SREBP1, FAS, ACC and PPARγ in rats [25]. In our study, it was observed that QPE caused a notable reduction in the mRNA levels of SREBP-1c, ACC and FAS (Figure 3a–c), alongside an elevation in the mRNA levels of CPT1 and PPARα, as opposed to the model group (Figure 3f,g). These results implied that QPE could effectively reduce intracellular lipid buildup by modulating the expression of genes that play a role in lipid metabolism. Notably, an elevated SREBP-2 mRNA expression level was measured in the steatosis state of HepG_2_ cells, implying that SREBP-2 may be involved in the induction of NAFLD (Figure 3d). In contrast to SREBP1, SREBP-2 mainly controls TC synthesis and can regulate the expression of proteins associated with cholesterol biosynthesis, including the important rate-limiting enzyme HMGCR. This study identified a substantial increase in the mRNA levels of SREBP-2 and HMGCR in the model group as opposed to untreated cells, possibly leading to increased TC synthesis (Figure 3d,e). Earlier research revealed that berberine regulated cholesterol synthesis by targeting the SREBP-2-HMGCR signal pathway [26]. It was consistent with our findings that mRNA levels of SREBP-2 and HMGCR were greatly reduced after QPE, suggesting that the decreased intracellular TC synthesis was likely mediated by regulating the expression of SREBP-2 and HMGCR.

AMPK signaling is one of the key factors regulating lipid homeostasis and is considered as an effective therapeutic target for NAFLD. According to a recent study, a substantial increase in the activity of the SREBP-1c/FAS pathway was exhibited in NAFLD mice [27]. Additionally, experiments have provided strong evidence for the connection between the suppression of the SREBP-1c/FAS pathway and a decline in hepatic lipid synthesis [28]. As reported, AMPK-dependent phosphorylation controls the protein degradation and nuclear localization of SREBP-1c [25], which further inhibits the expression of downstream target genes. In our investigation, QPE remarkably upregulated AMPK phosphorylation, while decreasing the expression of SREBP-1c and FAS in comparison to the FFA-only group (Figure 3h–k). Consistent with previous studies, the activation of AMPK inhibited the activity of SREBP-1c and further suppressed the transcription of DNL-related genes, thereby reducing adipogenesis and ultimately slowing down lipid accumulation [29]. These results imply an ameliorative effect of QPE on FFA-induced hepatic steatosis by modulating the AMPK/SREBP-1c signaling pathway.

FA was found to be one of the major polyphenols of QPE and was reported to possess various hepatoprotective, anti-inflammatory, antioxidant and anti-tumor effects [30]. Network pharmacology has become a new approach for investigating the effects and mechanisms of biologically active nutrients [31]. Therefore, we finally analyzed the potential targets and molecular mechanisms of FA for alleviating NAFLD by network pharmacology. The PPI network analysis showed that there were 37 nodes and 172 edges in the network of FA acting on NAFLD. Eight key genes were screened based on degree and betweenness: TP53, EGFR, MMP9, APP, MMP2, STAT3, IFNG and RELA. The interaction of FA with core proteins was predicted by molecular docking experiments. Smaller values of molecular binding energy indicate a tighter binding. A binding energy <−5.0 kcal·mol^−1^ was considered to indicate a strong binding of the ligand to the target site, and a binding energy <−7.0 kcal·mol^−1^ suggests a stronger affinity between the two [32]. The results showed that the three protein targets that bound most strongly to FA were MMP9, MMP2 and EGFR. FA formed hydrogen bonds with the PRO-246 and CYS-99 sites of MMP9 and the THR-248 site of MMP2 (Figure 6a,b). It was also noted that FA formed hydrogen bonds with four different sites of EGFR (Figure 6c). MMP-9 played an important role in promoting the formation of NAFLD and the progression of liver fibrosis [33]. One study reported that MMP9 could distinguish patients at risk of progression from non-alcoholic steatohepatitis to hepatocellular carcinoma [34]. It was also reported that EGFR may inhibit lipid accumulation and liver fibrosis through mechanisms involving the regulation of oxidative stress [35]. In addition, the GO analysis showed that targets in the PPI network were primarily associated with the regulation of inflammatory cytokines and gene expression, including IL-1β, IL-6, IL-8, IL-10, IL-12 and chemokine. Moreover, the KEGG enrichment analysis showed that FA mainly functioned in ROS, the HIF-1 signaling pathway, lipids and atherosclerosis, insulin resistance, and the PI3K-Akt signaling pathway. As previously mentioned, oxidative stress induced by ROS and inflammation is an important trigger of NAFLD. Our data provide evidence to further explore the above key targets and signaling pathways.

Taken together, the present study demonstrated that QPE effectively decreased lipid accumulation, reduced oxidative stress and alleviated inflammatory response in a steatosis cell model. The alleviating effect of QPE on NAFLD required AMPK activation to hamper the maturation of SREBP-1c and consequently decrease the lipid accumulation derived from DNL. These findings may offer valuable insights into how quinoa can alleviate hepatic steatosis and establish a theoretical basis for the integration of quinoa in functional food research and development. Notwithstanding the findings of this study, it is crucial to address its limitations. This is mainly due to the fact that the single cell line model (HepG_2_ cells) used in the study may pose a constraint on the generalizability of the results. In order to overcome this limitation, future studies could consider the inclusion of primary hepatocytes or multiple different hepatocyte cell lines. At the same time, an in vivo model could be introduced to validate the results of the in vitro study and further assess the bioavailability and metabolic pathways of QPE.

## 5. Conclusions

To conclude, this study sought to analyze the impact and molecular pathway through which QPE and its major phenolic acids alleviate NAFLD. Cellular experiments confirmed that QPE could effectively alleviate NAFLD from three angles: alleviating lipid accumulation, lowering oxidative stress markers and modulating inflammatory cytokine levels. Notably, the reduced intracellular TG synthesis was likely mediated by the activation of the AMPK/SREBP-1c signaling pathway. Furthermore, the application of network pharmacology helped anticipate the core targets and molecular pathways of FA in mitigating NAFLD. The enrichment analysis results indicated that the improvement mechanism of FA was closely related to the inhibition of oxidative stress, insulin resistance and the regulation of inflammatory cytokines. The overall results suggest that QPE is a potential dietary supplement in the treatment of NAFLD, as it can reduce oxidative stress, decrease hepatic inflammation and inhibit hepatic steatosis by activating the AMPK pathway. Nevertheless, additional studies are required to understand how QPE exerts beneficial effects in animal models of NAFLD and the regulatory mechanisms involved.

## Figures and Tables

**Table 1:** UPLC Q-TOF-MS analysis of polyphenolic components in QPE.

No.	Compound	RT (min)	[M−H]^−^ (*m*/*z*)	Fragment Ions (*m*/*z*)
1	Vanillic acid	4.50	167.0391	152.0116, 108.0229
2	Protocatechuic acid	4.53	153.0188	109.0297, 108.0218
3	Caffeic acid	5.48	179.0422	135.0458
4	*p*-Hydroxybenzoic acid	6.07	137.0302	93.0341
5	*p*-Coumaric acid	6.78	163.0401	119.0509
6	Ferulic acid	7.60	193.0555	178.0267, 149.0601, 134.0368
7	(-)-Catechin	8.06	289.0862	175.0351, 137.0212, 134.0440, 123.0479
8	(-)-Epicatechin	8.07	289.0862	137.0243, 123.0448, 109.0295
9	Sinapic acid	8.15	223.0689	194.0116, 165.0168, 147.0482

**Table 2:** Docking information of core targets with FA.

Targets	PDBID	Binding Energy (kcal·mol^−1^)
APP	1AAP	−5.2
EGFR	3POZ	−6.2
IFNG	3BES	−5.5
MMP2	1CK7	−6.4
MMP9	5UE3	−7.3
RELA	1NFI	−5.8
STAT3	6NJS	−5.8
TP53	2BIM	−5.0

## Data Availability

The data can be accessed on request.

## Supplementary Materials

The following supporting information can be downloaded at: https://www.mdpi.com/article/10.3390/nu16142276/s1, Table S1: Primer sequences used in RT-qPCR. Table S2: Content of compounds in QPE. Table S3: Targets with higher-than-average betweenness and degree in PPI network.
