# Supplementary material for: Quinoa Polyphenol Extract Alleviates Non-Alcoholic Fatty Liver Disease via Inhibiting Lipid Accumulation, Inflammation and Oxidative Stress
Source: Nutrients. 2024 Jul 15;16(14):2276. doi: 10.3390/nu16142276 (PMC11279623; doi:10.3390/nu16142276)
Supplement: Supplementary file 1 [file nutrients-16-02276-s001.zip › nutrients-3096097-supplementary.pdf]

Table S1: Primer sequences used in RT-qPCR.

Genes	Forward primer sequence (5′-3′)	Reverse primer sequence (5′-3′)
SREBP-1c	ACAGTGACTTCCCTGGCCTAT	GCATGGACGGGTACATCTTCAA
FAS	TCTGGTTCTTACGTCTGTTGC	CTGTGCAGTCCCTAGCTTTCC
ACC	ATGTCTGGCTTGCACCTAGTA	CCCCAAAGCGAGTAACAAATTCT
SREBP-2	CTGCAACAACAGACGGTAATGA	CCATTGGCCGTTTGTGTCAG
HMGCR	TGATTGACCTTTCCAGAGCAAG	CTAAAATTGCCATTCCACGAGC
CPT-1	TCAAGCCAGACGAAGAACATC	TGGTAGGAGAGCAGCACCTT
PPARα	ATGGAGACCTTGTGTATGG	ATCTGGATGGTTGCTCTG
IL-1β	AGCTACGAATCTCCGACCAC	CGTTATCCCATGTGTCGAAGAA
IL-6	ACTCACCTCTTCAGAACGAATTG	CCATCTTTGGAAGGTTCAGGTTG
IL-10	TCAAGGCGCATGTGAACTCC	GATGTCAAACTCACTCATGGCT
TNF-α	CCTCTCTCTAATCAGCCCTCTG	GAGGACCTGGGAGTAGATGAG
GAPDH	GGAGCGAGATCCCTCCAAAAT	GGCTGTTGTCATACTTCTCATGG

Table S2: Content of compounds in QPE.

compound	content (μg/g)
Protocatechuic acid	95.93±1.65
p-Hydroxybenzoic acid	6.19±0.53
Vanillic acid	3.95±0.05
Caffeic acid	4.70±0.46
p-Coumaric acid	1.07±0.15
Ferulic acid	58.17±1.84
Sinapic acid	1.97±0.66

Table S3: Targets with higher-than-average betweenness and degree in the PPI network.

Gene name	Betweenness	Degree
TP53	301.92	26.00
EGFR	190.45	23.00
MMP9	126.36	20.00
APP	76.80	15.00
MMP2	75.45	15.00
STAT3	64.67	20.00
IFNG	56.07	18.00
RELA	48.16	15.00
